# Medical Items

**Published:** 1894-09

**Authors:** 


### *MEDICAL ITEMS.*

---

THE twentieth annual meeting of the Mississippi Valley Medical Association will occur in Hot Springs, Ark., November 20, 21, 22 and 23, 1894. Frederick C. Woodburn, Secretary, Indianapolis, Ind.

---

DON'T forget the meeting of the Tri-State Medical Society in Atlanta, October 9th, 10th and 11th. Important business will come before the Society. A change of name will be discussed. Be on hand with a good paper. Send title of paper to Dr. Frank T. Smith, Chattanooga.

---

OHIO is about the most "doctored" State in the Union. Cincinnati has one physician to every 460 inhabitants, Cleveland one to every 500, and Columbus one to every 360. The reason is too well known to require comment. We present our condolements to our esteemed friend the *Lancet-Clinic.*

ELIZA WORTHINGTON, a colored woman living near Chattanooga, Tenn., recently gave birth to triplets, two *black* and one *white*. Three reputable physicians, who attended the woman, and some scores of private citizens, vouch for the truth of the story. Another argument for superfetation.—*Medical Age.*

---

THE officers of the American Medical Editors' Association for the ensuing year are: President, Doctor John B. Hamilton, of Chicago; Vice-President, Doctor G. M. Gould, of Philadelphia; Secretary, Doctor H. B. Ellis, of San Francisco. The next meeting will be held in Baltimore in May, 1895.

---

THE Medical and Surgical Society of Mississippi was organized at Jackson, May 20. One hundred and twenty prominent physicians enrolled their names. This organization is the outcome of dissensions in the State Medical Association. Dr. J. H. Lucas, of Greenville, is temporary President, and Dr. H. H. Hughes Secretary.

---

MEDICAL EDUCATION IN ST. LOUIS.—Dr. Albert Abrams, of San Francisco, in a late letter to the *Occidental Medical Times*, says:

St. Louis is a city of medical schools and free dispensaries. I counted as many as fourteen medical schools in St. Louis, and this list does not include many schools not reckoned under the caption of colleges in the directory. There is even a school of osteopathy established by a bone setter. * * Competition in medical education, thus engendered, is not exempt from the odium which accompanies the competition of trade. Even by a most charitable process of ratiocination, such medical colleges are naught else but mills, but unlike those of the gods, they grind quickly. I overheard the following conversation between two men in St. Louis. Said the one, "Hello, Bill! you ain't driving a car no more?" "No," said Bill, "it's too hard work; and, besides that, I'm going to become a doctor. It's a blamed sight easier to wear a plug hat and glasses than to drive a car. Doc. (referring to a professor in a regular school) said I'd get through all right if I paid the fees." This conversation I hope will not be accepted as an apt illustration of the status of medical education in St. Louis.

